# Online Crowdfunding for Urologic Cancer Care

**DOI:** 10.3390/cancers14174104

**Published:** 2022-08-25

**Authors:** Pawel Rajwa, Philip Hopen, Jakub Wojnarowicz, Julia Kaletka, Iga Paszkiewicz, Olga Lach-Wojnarowicz, Hadi Mostafaei, Wojciech Krajewski, David D’Andrea, Bartosz Małkiewicz, Andrzej Paradysz, Guillaume Ploussard, Marco Moschini, Benjamin N. Breyer, Benjamin Pradere, Shahrokh F. Shariat, Michael S. Leapman

**Affiliations:** 1 Department of Urology, Medical University of Vienna, 1090 Vienna, Austria; 2 Department of Urology, Medical University of Silesia, 41-800 Zabrze, Poland; 3 Data Science & Clinical Analytics, New Century Health, Boston, MA 02481, USA; 4 Department of Minimally Invasive and Robotic Urology, University Center of Excellence in Urology, Wroclaw Medical University, 50-556 Wroclaw, Poland; 5 Department of Urology, La Croix du Sud Hospital, 31130 Quint Fonsegrives, France; 6 Division of Oncology, Unit of Urology, Urological Research Institute, IRCCS Ospedale San Raffaele, 20132 Milan, Italy; 7 Department of Urology, University of California San Francisco, San Francisco, CA 94143, USA; 8 Department of Biostatistics and Epidemiology, University of California San Francisco, San Francisco, CA 94143, USA; 9 Institute for Urology and Reproductive Health, Sechenov University, 119435 Moscow, Russia; 10 Karl Landsteiner Institute of Urology and Andrology, 1090 Vienna, Austria; 11 Department of Urology, Weill Cornell Medical College, New York, NY 10021, USA; 12 Department of Urology, University of Texas Southwestern, Dallas, TX 75390, USA; 13 Department of Urology, Second Faculty of Medicine, Charles University, 15006 Prague, Czech Republic; 14 Hourani Center for Applied Scientific Research, Al-Ahliyya Amman University, Amman 19328, Jordan; 15 Department of Urology, Yale University School of Medicine, New Haven, CT 06520, USA

**Keywords:** urology, crowdfunding, prostate cancer, bladder cancer, kidney cancer, testicular cancer

## Abstract

**Simple Summary:**

Our goal was to analyze the financial needs of patients with urological cancer on the basis of online crowdfunding campaigns. In our report, we included 2126 individual campaigns fundraising for prostate, bladder, kidney and testicular cancer. We specified the detailed financial needs of uro-oncologic patients, campaigns’ characteristics and the factors associated with campaigns’ success and high fundraising. Our work showed that, with the constantly increasing costs of medical care, patients are looking for funding from online communities including seeking funds for alternative therapies. Overall, these data point to a wide spectrum of patient needs for urologic cancer care as well as identifiable factors influencing campaign success.

**Abstract:**

Background: we aimed to characterize the financial needs expressed through online crowdfunding for urologic cancers. Methods: the data used in this study came from the online crowdfunding platform GoFundMe.com. Using an automated software method, we extracted data for campaigns related to urologic cancers. Subsequently, four independent investigators reviewed all extracted data on prostate, bladder, kidney and testicular cancer. We analyzed campaigns’ basic characteristics, goals, fundraising, type of treatment and factors associated with successful campaigns. Results: in total, we identified 2126 individual campaigns, which were related to direct treatment costs (34%), living expenses (17%) or both (48%). Median fundraising amounts were greatest for testicular cancer. Campaigns for both complementary and alternative medicine (CAM) (median $11,000) or CAM alone (median $8527) achieved higher fundraising totals compared with those for conventional treatments alone (median $5362) (*p* < 0.01). The number of social media shares was independently associated with campaign success and highest quartile of fundraising. Conclusions: using an automated web-based approach, we identified and characterized online crowdfunding for urologic cancer care. These findings indicated a diverse range of patient needs related to urologic care and factors related to campaigns’ success.

## 1. Introduction

In recent years, online medical crowdfunding has become an increasingly popular tool to defray the financial toxicity associated with cancer care [1,2]. Present evidence supports a prominent role for crowdfunding in supplementation of cancer care costs and their broader impact on patients, caregivers and communities [1,2]. Initially adopted in countries without universal healthcare coverage, such as the United States, online crowdfunding has expanded worldwide and enable patients to raise funds for cancer therapies and indirect treatment-related expenses [3,4].

Urologic malignancies, including prostate, bladder, kidney and testicular cancer constitute approximately 16.5% of all cancer diagnoses and are responsible for 10.8% of all cancer-related deaths [5]. A wide variety of therapeutic options are appropriate for urologic cancers, including surveillance, radical or organ-sparing surgeries, chemo, radiation, immunotherapy, as well as investigational or experimental approaches. Therefore, the spectrum of personal and financial hardships associated with their diagnosis may vary by cancer type and manner of treatment [6,7]. Due to growing interests in complementary and alternative medicine (CAM), the true scale of financial burdens associated with urologic cancer remains incompletely defined [1,2]. Furthermore, while it is apparent that cancer diagnosis has a direct impact on physical and mental health, the financial toxicity of cancer care cannot be underestimated [8,9,10]. The increased risk of bankruptcy, loss of work-related income, and un- and underinsurance may delay or even prevent care-seeking of needy cancer patients, leading to higher morbidity and mortality [7,8]. Thus, many urologic cancer patients and their families seek assistance for financial needs during cancer treatment through a variety of means.

Up until now, there has been only sparse data analyzing online medical crowdfunding in urologic oncology [11,12]. Hence, through large-scale analysis of online crowdfunding campaigns, we aimed to focus on crowdfunding for urologic cancer care and related expenses. Our objective was to characterize online crowdfunding campaigns for urologic cancer and to evaluate financial needs, campaign characteristics, goals and factors associated with success.

## 2. Materials and Methods

This study was based on data from GoFundMe.com, the largest online crowdfunding platform. We performed data extraction using an automated web browser (Selenium, https://www.selenium.dev/) to identify English-language campaigns for common urologic cancers (prostate, bladder, kidney, and testicular) and extracted the hyperlink address. Next, we exported text from each campaign using a web-based tool (scrapy.org) [4]. Four independent investigators (J.W., J.K., O.P., O.L.W.) subsequently performed a manual search to exclude campaigns fundraising for charities, animals, events, research, and other cancers and analyzed the campaigns’ descriptions to determine direct needs and patient characteristics. We categorized complementary and alternative medicine (CAM) as described previously [2]. Disagreements were resolved at the authors’ consensus meeting. Next, within a three-day period, we reevaluated ongoing campaigns to exclude deactivated ones and updated the figures to mitigate the time differences of manual analysis.

Association between campaigns’ characteristics and cancer types or type of treatments were assessed using the Pearson’s Chi-squared test, Fisher’s exact test or Kruskal–Wallis rank sum test, as appropriate [13,14]. Univariable and multivariable logistic regression analyses were to performed to evaluate factors associated with successful campaigns (defined as reaching ≥100% of the fundraising goal) and highest fundraisers (a highest quartile dollar amount). We used Firth correction to mitigate the bias associated with the disbalance between dependent variable groups [15]. Models’ discrimination was tested with the area under the curve (AUC) derived from the receiver operating (ROC) curve. Analyses were performed using R Version 4.2 (R Foundation for Statistical Computing, Vienna, Austria, 2022).

## 3. Results

**Figure 1:**
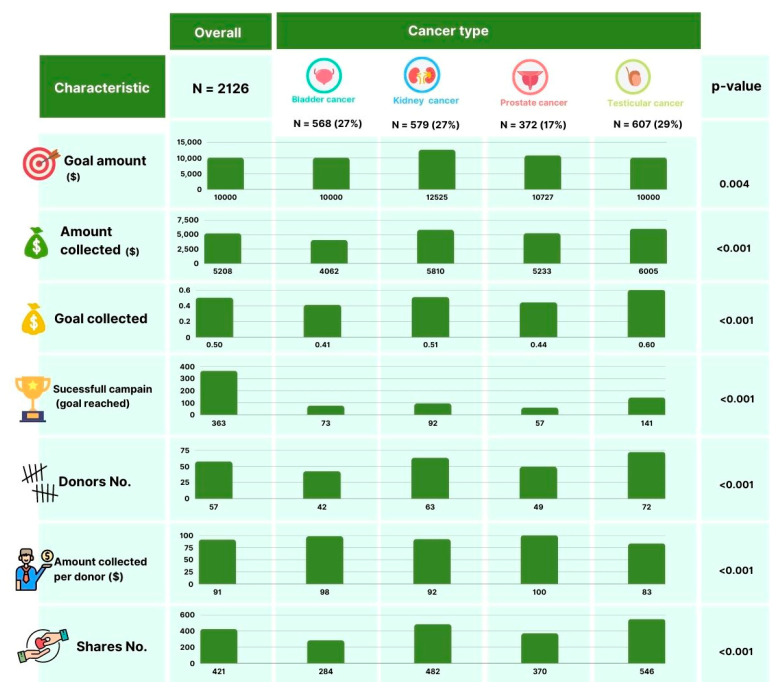
Selected figures of 2126 GoFundMe.com online crowdfunding campaigns fundraising for urologic malignancies stratified by cancer type.

The final study cohort included 2126 (67.88% of initially extracted) eligible campaigns (Table 1, Figure 1). The total requested amount was $46,369,821, and $19,879,097 was collected during a median of 920 (IQR 573–1336) days since the campaign was initiated. Most campaigns originated from the US (95%). The primary needs expressed by campaigns were direct treatment costs (34%), living expenses (e.g., transportation, lost wages, nonmedical bills) (17%) or both (48%). The median fundraised totals differed by cancer type: testicular cancer $6005 (IQR 3285–11,090), kidney cancer $5810 (IQR 2628–13,272), prostate cancer $5233 (IQR 2171–12,255) and bladder cancer $4062 (IQR 2284–8570) (*p* < 0.01). Campaigns for testicular cancer more frequently reached their stated fundraising goal (23%), had the highest median number of social media shares (median 546 [IQR 320–976]), and had the most donors (median 72 [IQR 43–125]), but the lowest amount collected per donor (median $83 [IQR 66–109]) (all *p* < 0.01).

**Figure 2:**
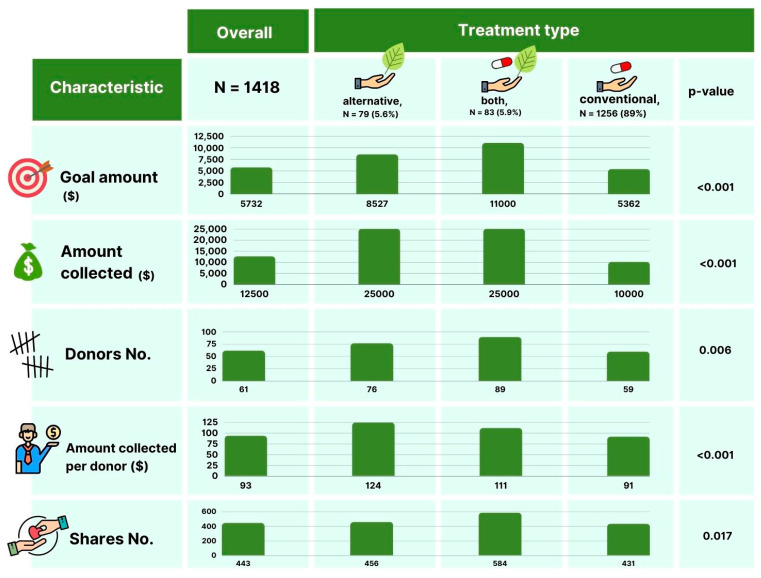
Selected figures of 1418 GoFundMe.com online crowdfunding campaigns fundraising for urologic malignancies stratified by treatment type.

We further compared fundraising for campaigns directed at CAM alone (5.6%), both CAM and conventional treatments (5.9%), and conventional treatments only (89%) (Table 2, Figure 2) [2]. Campaigns for CAM, alone or in combination, were more common in prostate cancer (22% and 12%, respectively), and more frequently originated in countries outside of the US (13% vs. 4%; *p* = 0.003). Campaigns for CAM, alone or in combination with conventional treatments, had higher fundraising goals compared to those for conventional treatment (median: $25,000 [IQR 15,750–40,000] vs. $25,000 [IQR 12,500–50,000] vs. $10,000 [IQR 7000–25,000], respectively; *p* < 0.001) and had higher fundraising totals ($8527 [IQR 4762–16,206] vs. $11,000 [IQR 4830–20,702] vs. $5362 [IQR 2708–11,334], respectively; *p* < 0.001). CAM-directed campaigns had a higher number of social media shares compared with conventional therapy alone (median 456 [IQR 177–712] vs. 584 [IQR 292–938) vs. 431 [IQR 236–806]; *p* = 0.017), a higher amount collected per donor ($124 [IQR 99–195] vs. $111 [IQR 86–156] vs. $91 [IQR 69–119]; *p* = 0.006) and per social media share ($23 [IQR 13–35] vs. $19 [IQR 9–32] vs. $13 [IQR 7–24]; *p* < 0.001) for CAM-alone, CAM and conventional therapy and conventional therapy alone, respectively. There were no differences in terms of disease advancement (nonlocalized: 87% vs. 94% vs. 84%; *p* = 0.09). Campaigns fundraising for alternative/conventional treatment were more often started outside the US (13% vs. 4%; *p* = 0.003).

In multivariable analysis, social media shares (odds ratio [OR] per 100 shares: 1.03, 95% confidence interval [CI] 1.01–1.05, *p* < 0.001) and campaigns for testicular cancer (OR 2.03, 95% CI 1.34–3.11, *p* < 0.001) were associated with completion of fundraising goals (Table 3). Factors associated with high fundraising campaigns (1st quartile; >$11,300) included social media shares (OR per 100 shares 1.13, 95% CI, 1.10–1.16, *p* < 0.001), initial description length (OR per 100 words 1.08, 95% CI 1.02–1.14, *p* = 0.004), collecting for CAM alone (OR 2.56, 95% CI 1.33–4.99, *p* = 0.005), CAM and conventional treatment (OR 2.15, 95% CI 1.22–3.79, *p* = 0.009), and nonlocalized disease (OR 2.37, 95% CI 1.38–4.06, *p* = 0.001).

## 4. Discussion

In this study, we evaluated the current landscape of online crowdfunding for urologic cancer care using an automated approach to evaluate campaigns on GoFundMe.com. With growing recognition of the financial toxicities encountered during cancer diagnosis and treatment, particularly in the US healthcare system, this study highlighted the emergence of novel strategies enabled through enhanced social connectivity. There were several key findings of our study. First, while there were significant differences between campaigns directed at different urologic cancer types, testicular cancer campaigns were most likely to be successful. Second, campaigns fundraising for CAM, compared to conventional treatment, had higher fundraising totals and collected more per donor. Third, the number of social media shares was an independent predictor of the highest fundraising and campaign success.

These findings built on the work of other publications that have examined crowdfunding in urology [12,16,17]. For example, Di Carlo et al. analyzed 119 urology campaigns from Canada and found that urologic oncology campaigns fundraised more than campaigns raising for urologic benign conditions [12]. Notably, nine campaigns fundraising for testicular diseases were evaluated (all for benign conditions; none for testicular cancer) and these had the highest amount collected, similarly to our findings. Moreover, in another study, campaigns for prostate cancer were shown to collect significantly less than those for breast cancer [16]. Thomas et al. evaluated 486 GoFundMe^®^ kidney campaigns and found that among 486 kidney cancer campaigns, only 8% were successful; the median goal was $10,000, the median amount raised was $1450 and the median number of donors was 17 [17]. We identified considerable heterogeneity in the amount of funding requested and raised by cancer type. Campaigns for testicular cancer were the most successful in terms of dollar amount raised, a finding that may reflect the effect of the younger age and broader social networks of these patients. Indeed, younger patients with cancer face greater financial toxicity due to comparatively fewer savings, higher rates of uninsurance and potential impacts on family members and dependents [11,18].

We also found that a greater number of social media shares were associated with measures of a campaign’s success. These findings underscored the extent to which greater dissemination of campaigns may be an important component of successful fundraising. Our results expanded upon findings from previous studies examining the role of social media shares in online medical crowdfunding. For example, in a study of Fong et al., social media shares were correlated with amount raised [19]. Through analysis of 1100 GoFundMe campaigns directed at gender-affirming surgery, Akiki et al., determined that social media shares were associated with highest fundraising totals [20]. These results were also in agreement with an expanding body of work that has shown the role of social media in the promotion of other aspects of urologic care delivery, including the dissemination of novel research [21].

Lastly, we found that a large proportion of campaigns included fundraising for CAM treatments. Although there has been expanding interest in the use of these approaches [2], less has been known about the role of crowdfunding, given that they are frequently not covered by insurance. We further uncovered that campaigns directed at CAM treatments were more successful by dollar amount, a finding that could reflect an awareness of greater unmet financial needs or greater public enthusiasm for these treatments. These findings unmasked the rise in the usage of CAM among patients with urologic cancers, particularly as their use as stand-alone therapy has been associated with worse cancer outcomes [22]. Finally, limitations of this study included the cross-sectional design and an emphasis on campaigns originating in the United States. Other limitations included disbalance of the dependent variable and lack of data on fundraisers’ age. Future work could examine globally-directed crowdfunding campaigns.

## 5. Conclusions

To conclude, in this study, using a new novel web-based methodology analysis, we identified and characterized patients’ reported financial needs for urologic cancer care. Notably, while the majority of campaigns gathered money for medical bills, nearly two-thirds aimed at collecting funds for daily living costs, which were main or additional goals. Furthermore, our findings indicated a diverse range of online crowdfunding campaigns, their outcomes and identifiable factors associated with campaigns’ success. We showed that, while there were significant differences across campaigns fundraising for urologic cancers, testicular cancer initiatives were the most likely to succeed. Additionally, campaigns soliciting money for CAM had greater fundraising totals and raised more than efforts seeking money for conventional therapy. Finally, the number of social media shares was associated with successful campaign financing. Future research could examine crowdfunding efforts on a global basis.

## Figures and Tables

**Table 1:** Characteristics of 2126 GoFundMe.com online crowdfunding campaigns fundraising for urologic malignancies stratified by cancer type.

Characteristic	Overall	Cancer Type				*p*-Value
	N = 2126	Bladder, N = 568 (27%)	Kidney, N = 579 (27%)	Prostate, N = 372 (17%)	Testicular, N = 607 (29%)	
Campaigns from US	2016 (95%)	527 (93%)	557 (96%)	362 (97%)	570 (94%)	0.005
Goal amount ($)	10,000 (5000–25,000)	10,000 (5000–20,000)	12,525 (7500–25,000)	10,727 (5000–25,000)	10,000 (5500–20,000)	0.004
Amout collected ($)	5208 (2565–11,315)	4062 (2284–8570)	5810 (2628–13,272)	5233 (2171–12,255)	6005 (3285–11,090)	<0.001
% Goal collected	0.50 (0.24–0.84)	0.41 (0.19–0.73)	0.51 (0.24–0.83)	0.44 (0.19–0.80)	0.60 (0.33–0.95)	<0.001
Sucessfull campain (goal reached)	363 (17%)	73 (13%)	92 (16%)	57 (15%)	141 (23%)	<0.001
Campaign days running	920 (573–1336)	926 (589–1342)	870 (556–1302)	835 (553–1271)	1018 (619–1384)	0.001
Amount collected per day ($)	6 (3–12)	5 (2–9)	6 (3–15)	6 (2–14)	6 (3–12)	<0.001
Donors No.	57 (31–111)	42 (24–80)	63 (34–137)	49 (22–106)	72 (43–125)	<0.001
Amount collected per donor ($)	91 (69–122)	98 (73–127)	92 (69–120)	100 (69–139)	83 (66–109)	<0.001
Shares No.	421 (208–781)	284 (152–554)	482 (246–863)	370 (137–686)	546 (320–976)	<0.001
Amount collected per share ($)	13 (7–26)	14 (8–27)	13 (7–27)	16 (9–30)	12 (7–21)	<0.001
Followers No.	60 (32–115)	43 (26–84)	64 (36–138)	51 (23–109)	76 (46–127)	<0.001
Updates No.	2 (0–5)	2 (0–5)	2 (0–7)	2 (0–5)	2 (0–6)	0.3
Description length (words)	248 (159–392)	234 (148–360)	249 (160–418)	254 (156–427)	255 (168–382)	0.051
Female	360 (17%)	165 (29%)	195 (34%)	0 (0%)	0 (0%)	<0.001
Histology provided	379 (18%)	94 (17%)	165 (28%)	30 (8.1%)	90 (15%)	<0.001
First-person description	245 (12%)	47 (8.3%)	63 (11%)	61 (16%)	74 (12%)	0.002
Religious references	878 (41%)	220 (39%)	258 (45%)	163 (44%)	237 (39%)	0.10
Children mentions	972 (46%)	310 (55%)	264 (46%)	206 (55%)	192 (32%)	<0.001
Relationship status provided	930 (44%)	274 (48%)	213 (37%)	177 (48%)	266 (44%)	<0.001
Job status provided	985 (46%)	274 (48%)	253 (44%)	130 (35%)	328 (54%)	<0.001
Family status provided	1602 (75%)	385 (68%)	487 (84%)	258 (69%)	472 (78%)	<0.001
Photos No.	1.00 (1.00–3.00)	1.00 (1.00–3.00)	1.00 (1.00–3.00)	2.00 (1.00–4.00)	1.00 (1.00–2.00)	<0.001
Family photo provided	1071 (50%)	281 (49%)	287 (50%)	189 (51%)	314 (52%)	0.8
Un-/underinsurance mentions	623 (29%)	180 (32%)	165 (28%)	131 (35%)	147 (24%)	0.001
Fundraising for treatment	703 (34%)	140 (25%)	219 (41%)	134 (36%)	210 (36%)	<0.001
Fundraising for treatment & living expenses	985 (48%)	290 (52%)	251 (47%)	169 (46%)	275 (47%)	0.13
Fundraising only for living expenses	358 (17%)	124 (22%)	67 (12%)	68 (18%)	99 (17%)	<0.001
No data/Not applicable	80					
Fundrising for alternative treatment	79 (5.6%)	22 (6.1%)	3 (0.8%)	52 (22%)	2 (0.4%)	<0.001
Fundraising for conventional treatment	1256 (89%)	312 (87%)	356 (94%)	153 (65%)	435 (97%)	<0.001
Fundraising for conventional & alternative treatment	83 (5.9%)	25 (7.0%)	19 (5.0%)	29 (12%)	10 (2.2%)	<0.001
No data/Not applicable	708					
Localized disease	183 (14%)	57 (20%)	72 (19%)	27 (12%)	27 (6.1%)	<0.001
Nonlocalized disease	1147 (86%)	232 (80%)	306 (81%)	192 (88%)	417 (94%)	<0.001
No data on cancer advancement	796					
Fundraising for surgery	977 (58%)	260 (60%)	293 (62%)	86 (28%)	338 (70%)	<0.001
Fundraising for chemotherapy	851 (50%)	234 (54%)	161 (34%)	79 (26%)	377 (78%)	<0.001
Fundraising for radiation therapy	254 (15%)	53 (12%)	87 (19%)	80 (26%)	34 (7.0%)	<0.001
Fundraising for immunotherapy	109 (6.5%)	49 (11%)	49 (10%)	8 (2.6%)	3 (0.6%)	<0.001
n (%); Median (IQR)						

Abbreviations: IQR, interquartile range; n, number.

**Table 2:** Characteristics of 1418 GoFundMe.com online crowdfunding campaigns fundraising for urologic malignancies stratified by treatment type.

Characteristic	Overall	Treatment Type			*p*-Value
	N = 1418	Alternative, N = 79 (5.6%)	both, N = 83 (5.9%)	Conventional, N = 1256 (89%)	
Cancer Type					<0.001
Bladder	359 (25%)	22 (28%)	25 (30%)	312 (25%)	
Kidney	378 (27%)	3 (3.8%)	19 (23%)	356 (28%)	
Prostate	234 (17%)	52 (66%)	29 (35%)	153 (12%)	
Testicular	447 (32%)	2 (2.5%)	10 (12%)	435 (35%)	
Campaigns from US	1351 (95%)	76 (96%)	72 (87%)	1203 (96%)	0.003
Goal amount ($)	12,500 (7500–25,000)	25,000 (15,750–40,000)	25,000 (12,500–50,000)	10,000 (7000–25,000)	<0.001
Amount collected ($)	5732 (2845–12,102)	8527 (4762–16,206)	11,000 (4830–20,702)	5362 (2708–11,334)	<0.001
% Goal collected	48 (23–83)	41 (17–72)	41 (19–75)	49 (24–84)	0.038
Successful campain (goal reached)	231 (16%)	6 (7.6%)	9 (11%)	216 (17%)	0.031
Campaing days running	944 (581–1348)	1049 (685–1378)	1025 (670–1306)	928 (571–1348)	0.3
Amount collected per day ($)	6 (3–13)	9 (6–18)	10 (5–25)	6 (3–12)	<0.001
Donors No.	61 (33–115)	76 (38–124)	89 (50–143)	59 (32–113)	0.006
Amount collected per donor ($)	93 (70–125)	124 (99–195)	111 (86–156)	91 (69–119)	<0.001
Shares No.	443 (236–813)	456 (177–712)	584 (292–938)	431 (236–806)	0.017
Amount collected per share ($)	13 (7–26)	23 (13–35)	19 (9–32)	13 (7–24)	<0.001
Followers No.	63 (35–117)	76 (39–118)	86 (51–146)	61 (34–116)	0.023
Updates No.	2.0 (1.0–6.0)	3.0 (1.0–6.5)	2.0 (1.0–6.5)	2.0 (1.0–6.0)	0.2
Description lenght	276 (180–424)	317 (223–584)	359 (208–534)	268 (178–413)	<0.001
Female	238 (17%)	7 (8.9%)	14 (17%)	217 (17%)	0.2
Histology provided	288 (20%)	9 (11%)	15 (18%)	264 (21%)	0.10
First-person description	180 (13%)	17 (22%)	19 (23%)	144 (11%)	<0.001
Religious references	597 (42%)	38 (48%)	38 (46%)	521 (41%)	0.4
Children mentions	613 (43%)	36 (46%)	50 (60%)	527 (42%)	0.005
Relationship status provided	611 (43%)	38 (48%)	44 (53%)	529 (42%)	0.10
Job status provided	665 (47%)	30 (38%)	28 (34%)	607 (48%)	0.009
Family status provided	1050 (74%)	49 (62%)	69 (83%)	932 (74%)	0.009
Photos No.	1 (1–3)	2.00 (1–5)	2 (1–4)	1 (1–3)	0.004
Family photo provided	695 (49%)	35 (44%)	46 (55%)	614 (49%)	0.4
Un-/uninsurance mention	500 (35%)	42 (53%)	42 (51%)	416 (33%)	<0.001
Fundraising only for treatment	591 (42%)	50 (63%)	44 (53%)	497 (40%)	<0.001
Fundraising for treatment and living expenses	827 (58%)	29 (37%)	39 (47%)	759 (60%)	<0.001
Localized disease	144 (15%)	7 (13%)	4 (6.2%)	133 (16%)	0.089
Nonlocalized disease	802 (85%)	47 (87%)	61 (94%)	694 (84%)	0.089
Fundraising for surgery	977 (69%)	0 (0%)	42 (51%)	935 (74%)	<0.001
Fundraising for chemotherapy	851 (60%)	0 (0%)	40 (48%)	811 (65%)	<0.001
Fundraising for radiation therapy	254 (18%)	0 (0%)	17 (20%)	237 (19%)	<0.001
Fundraising for immunotherapy	109 (7.7%)	0 (0%)	17 (20%)	92 (7.3%)	<0.001
n (%); Median (IQR)					

Abbreviations: IQR, interquartile range; n, number.

**Table 3:** Uni- and multivariable logistic regression analyses for campaigns reaching goal amount (successful campaign) and highest fundraising campaigns. Bold numbers were significant.

**Campaigns Reaching Goal Amount (Successful Campaigns)**						
**Characteristic**	**Univariable Analysis**			**Multivariable Analysis**		
	**OR**	**95% CI**	***p*-Value**	**OR**	**95% CI**	***p*-Value**
Cancer						
Bladder	Ref.	Ref.		Ref.	Ref.	
Kidney	1.28	0.92–1.78	0.144	1.37	0.88–2.15	0.161
Prostate	1.23	0.84–1.78	0.281	1.41	0.84–2.34	0.189
Testicular	2.05	1.51–2.80	**<0.001**	2.03	1.34–3.11	**<0.001**
Description (per 100 words)	0.96	0.92–1.01	0.010	-		
Shares (per 100)	1.02	1.01–1.03	**0.003**	1.03	1.01–1.05	**<0.001**
Updates	1.01	1.00–1.01	0.254	-		
Days running (per 100)	1.02	0.99–1.04	0.167	-		
Religious references	0.73	0.57–0.92	**0.007**	0.79	0.58–1.06	0.118
Treatment						
Conventional	Ref	Ref.		Ref.		
Alternative	0.43	0.17–0.89	**0.022**	0.51	0.20–1.13	0.099
Both	0.61	0.29–1.16	0.142	0.76	0.35–1.47	0.431
Children mentions	0.69	0.55–0.87	**0.002**	0.95	0.68–1.34	0.787
Family status	0.75	0.59–0.97	**0.028**	0.78	0.54–1.12	0.175
Job status	1.11	0.89–1.39	0.366	-		
Un-/underinsurance	0.70	0.53–0.90	**0.006**	0.83	0.61–1.14	0.251
Provided data on cancer advancement	0.78	0.62–0.98	**0.032**	0.75	0.55–1.02	0.067
Nonlocalized cancer	1.25	0.81–2.02	0.317	-		
Provided data on cancer histology	0.82	0.60–1.11	0.195	-		
				Model AUC 0.628		
**Campaigns Reaching Goal Amount (Higest Quartile; >11,310$)**						
	**Univariable Analysis**			**Multivariable Analysis**		
**Characteristic**	**OR**	**95% CI**	***p*-Value**	**OR**	**95% CI**	***p*-Value**
Cancer						
Bladder	Ref.	Ref.				
Kidney	1.92	1.46–2.54	**<0.001**	1.28	0.80–2.07	0.294
Prostate	1.84	1.36–2.51	**<0.001**	0.96	0.56–1.63	0.871
Testicular	1.45	1.10–1.93	**0.009**	0.98	0.62–1.57	0.948
Description (per 100 words)	1.14	1.10–1.18	**<0.001**	1.08	1.02–1.14	**0.004**
Shares (per 100)	1.13	1.11–1.15	**<0.001**	1.13	1.10–1.16	**<0.001**
Updates	1.03	1.02–1.04	**<0.001**	1.00	0.98–1.02	0.823
Days running (per 100)	1.04	1.02–1.06	**<0.001**	1.01	0.98–1.04	0.595
Religious references	1.02	0.84–1.25	0.82	-		
Treatment						
Conventional	Ref.	Ref.				
Alternative	2.04	1.27–3.23	**0.003**	2.56	1.33–4.99	**0.005**
Both	2.91	1.86–4.55	**<0.001**	2.15	1.22–3.79	**0.009**
Children mentions	1.13	0.92–1.37	0.237	-		
Family status	1.49	1.17–1.91	**0.001**	1.36	0.92–2.04	0.119
Job status	1.07	0.88–1.30	0.512	-		
Un-/underinsurance	0.90	0.72–1.11	0.332	-		
Nonlocalized disease	2.40	1.57–3.69	**<0.001**	2.37	1.38–4.06	**0.001**
Provided data on cancer histology	1.13	0.88–1.45	0.342	-		
				Model AUC: 0.764		

Abbreviations: CI: Confidence Interval; OR: Odds Ratio; Ref.: Reference.

## Data Availability

The data presented in this study are available on request from the corresponding author. The data are not publicly available due to privacy restrictions.
